# Editorial: Olfactory dysfunction in neurodegenerative diseases

**DOI:** 10.3389/fnins.2025.1611870

**Published:** 2025-05-08

**Authors:** Debanjan Dasgupta, Thomas Heinbockel, Swagata Ghatak

**Affiliations:** ^1^Neural Circuit Dynamics Lab, Department of Biological Science and Engineering, Indian Institute of Technology, Kanpur, India; ^2^Department of Anatomy, Howard University College of Medicine, Washington, DC, United States; ^3^School of Biological Sciences, National Institute of Science Education and Research, Bhubaneswar, India

**Keywords:** neurodegeneration, olfaction, diagnosis, Alzheimer's disease, Parkinson's disease

Neurodegenerative disorders are a major health concern in the aging world. Olfactory dysfunction (OD) is a condition characterized by altered olfactory perception. OD has been observed to be associated with many disorders, such as Parkinson's Disease (PD) and Alzheimer's Disease (AD). The OD is evident much earlier than the diagnosis of cognitive decline. Over the last few decades, a connection between OD and clinical symptoms of neurodegenerative diseases has been observed in studies (Marin et al., 2018). According to one study, 90% of PD cases show OD in the early stages (Doty, 2012), while 80% of AD patients show a notable decrease in their ability to smell (Zou et al., 2016). However, little is known about the mechanisms that can causally link the observed OD in different neurodegenerative disorders. The current Research Topic is a collection of research articles surrounding this central idea.

Olfactory stimulation takes place either ortho-nasally, such as during sniffing, or retro-nasally, such as when olfactory receptors get activated by odors coming from the mouth during food consumption (Whitcroft and Hummel, 2020). The first route helps more in ambient odor perception, while the latter route plays a role in flavor detection (Small, 2012). Retro-nasal olfactory stimulation should not be confused with taste, which takes place in taste buds in the oral cavity. Depending on the symptoms, OD is classified into anosmia (total loss of the sense of smell), hyposmia (diminished capacity to detect odors), phantosmia (perception of odors without an actual source present), and parosmia (misinterpretation of odors) (Hummel et al., 2023). These can be grouped into qualitative (parosmia and phantosmia) and quantitative (anosmia and hyposmia) dysfunctions. These two types of OD can occur in isolation or in combination.

A variety of conditions can cause ODs. Broadly, they can be classified into age-related, acquired, or congenital ODs. Infections in the upper respiratory tract, like those from COVID-19, can also cause OD. Other than this, most of the sinonasal inflammations cause a temporary OD during and post-infection. Neurodegenerative disorders are the next major cause for OD. Furthermore, ODs associated with neurodegenerative disorders are not temporary and rather deteriorate with the progression of the disorder. Furthermore, OD in cognitively healthy people could indicate preclinical neurodegenerative disorders (Marin et al., 2018).

The current Research Topic has a collection of review articles encircling the correlation of OD with neurodegenerative disorders (De Cleene et al.; Chen and Kostka), especially AD. Based on reports from WHO, nearly 57 million people suffer from dementia worldwide, with an increasing rate of ~18% annually. Out of this, more than 70% of the cases are due to AD. The early stage of AD is associated with subtle cognitive decline, which is usually within the clinical limits of normalcy (Jessen et al., 2014). Reports from olfactory behavioral testing and fMRI-based approaches have demonstrated the strong link of OD with early stages of AD. Review articles from Elhabbari et al., Liu et al., and Jeffs et al., summarize the connection in the topic. The review by Jeffs et al. includes a different dimension of circadian dysfunction associated with AD. Indeed, circadian rhythm dysfunction has been reported to be highly prevalent in AD patients (Leng et al., 2019). However, dysfunction in both circadian rhythm and olfaction has been associated with different neurodegenerative disorders and normal aging, a combinatorial approach suggested by Jeffs et al. that might allow the detection of neurodegenerative disorders categorically. This might be because of the commonality of the association of the glymphatic system, a macroscopic waste clearance system in the central nervous system, with AD (Murdock et al., 2024) as well as sleep (Hablitz et al., 2020).

Overall, this topic provides up-to-date information regarding the link between olfactory dysfunction and some of the major neurodegenerative disorders experienced by humankind. Furthermore, it is a timely attempt to raise the awareness of the neuroscience community to think toward strategies that can help in detection, delay, and reversal of such devastating disorders.
